# Recombinant expression and functional analysis of proteases from *Streptococcus pneumoniae, Bacillus anthracis, and Yersinia pestis*

**DOI:** 10.1186/1471-2091-12-17

**Published:** 2011-05-05

**Authors:** Keehwan Kwon, Jeremy Hasseman, Saeeda Latham, Carissa Grose, Yu Do, Robert D Fleischmann, Rembert Pieper, Scott N Peterson

**Affiliations:** 1 Pathogen Functional Genomics Resource Center, J. Craig Venter Institute, 9704 Medical Center Drive, Rockville, Maryland 20850, USA

## Abstract

**Background:**

Uncharacterized proteases naturally expressed by bacterial pathogens represents important topic in infectious disease research, because these enzymes may have critical roles in pathogenicity and cell physiology. It has been observed that cloning, expression and purification of proteases often fail due to their catalytic functions which, in turn, cause toxicity in the *E. coli *heterologous host.

**Results:**

In order to address this problem systematically, a modified pipeline of our high-throughput protein expression and purification platform was developed. This included the use of a specific *E. coli *strain, BL21(DE3) pLysS to tightly control the expression of recombinant proteins and various expression vectors encoding fusion proteins to enhance recombinant protein solubility. Proteases fused to large fusion protein domains, maltosebinding protein (MBP), SP-MBP which contains signal peptide at the N-terminus of MBP, disulfide oxidoreductase (DsbA) and Glutathione S-transferase (GST) improved expression and solubility of proteases. Overall, 86.1% of selected protease genes including hypothetical proteins were expressed and purified using a combination of five different expression vectors. To detect novel proteolytic activities, zymography and fluorescence-based assays were performed and the protease activities of more than 46% of purified proteases and 40% of hypothetical proteins that were predicted to be proteases were confirmed.

**Conclusions:**

Multiple expression vectors, employing distinct fusion tags in a high throughput pipeline increased overall success rates in expression, solubility and purification of proteases. The combinatorial functional analysis of the purified proteases using fluorescence assays and zymography confirmed their function.

## Background

Proteases represent one of the largest protein families, and play critical roles in cellular functions and viability in all organisms [1,2]. Proteases have diverse biological roles in signal transduction, post-translational modification, proliferation, apoptosis and pathogenicity through specific processing or non-specific degradation [3-6]. Proteases can be classified into two categories, secreted proteases and intracellular proteases [7]. Secreted proteases such as trypsin, usually cleave at specific short recognition sites of proteins or peptides. In contrast, substrates of intracellular proteases are much more specific, preventing uncontrolled degradation of cellular compartments. Major functions of intracellular proteases include clearing damaged proteins and playing roles as a part of regulatory pathways through the degradation of specific substrates [7-9]. For example, ClpXP in *Y. pestis *and Lon proteases contribute to the environmental regulation of the *Y. pestis *T3SS system through regulated proteolysis of YmoA [10]. Another example is a prenyl-protein-specific endoprotease that is involved in the post-translational modification processing steps of CAAX motif proteins [11]. Proteases also play key roles in the immune system for both defense mechanisms of host cells and pathogenicity in a variety of pathogens from viruses to higher parasites [11-16]. For example, IgA protease, an essential protein of *S. pneumoniae *in lung infection, targets a host immune response, secretory IgA [17]. Proteases of pathogens are potential therapeutic targets and therefore an understanding of their mechanisms and the discovery of new proteases are also important for defining novel drug targets [18-20].

Proteases can be also categorized as exopeptidases and endopeptidases [21]. Exopeptidases cleave peptide bonds at either the amino or carboxyl termini and sequentially hydrolyze amino acids. Endopeptidases cleave peptide bonds within the proteins. Endopeptidases may be further sub-divided into four types according to their catalytic mechanism: serine, cysteine, aspartic and metallo proteases. Serine proteases recognize specific cleavage site through their specificity pockets and catalyze the peptide bond cleavage using a conserved catalytic triad consisting of histidine, serine and aspartate [22]. Cysteine proteases use a similar catalytic triad as serine proteases except that a cysteine residue is recognized instead of serine. Aspartic proteases use two acidic residues in the catalytic process and metalloproteases use a metal ion and a glutamic acid to catalyze proteolysis both using a water molecule to cleave the peptide bond directly. Metalloproteases are comprised of 51 families and half of them fall into 3 clans, MA, MB and MX/MBA. They have common zinc binding motif, HEXXH and an additional zinc coordinating residue [23-25].

Due to the catalytic activity and biological consequences of peptide bond cleavage, proteases represent one of the most challenging functional groups of proteins for heterologous expression and purification. Production of unregulated foreign proteases in *E. coli *host cells represents a critical stress often resulting in the formation of inclusion bodies, non-expression, or cytotoxicity [26-28]. Therefore, the large-scale study of proteases is challenging. According to a previous statistical analysis in genome-wide expression and purification of *S. pneumoniae *proteins using an expression vector pHis, success frequencies of proteases were 37% for cloning, and only15% for recovery of soluble expressed recombinant protein [29].

The fusion tags used in the expression and purification of recombinant proteins are crucial components. Numerous tags have been utilized in detection, quantification, enhancement of expression and solubility, immobilization and purification of proteins [30-35]. The His-tag, maltose-binding protein (MBP), glutathione S-transferase (GST), thioredoxin (Trx) and the transcription termination anti-termination factor (NusA) are the most frequently used fusions tag. The His-tag and GST are widely used for affinity purification. These tags can also be used for detection of recombinant proteins and immobilization [30,36]. S-tag and green fluorescence protein (GFP) are used for detection and quantification of proteins [37,38]. Beside those fusion tags, Halo-tag is a more recently developed fusion tag for affinity purification, detection, immobilization and enhancing solubility of proteins [39]. The large fusion tags such as Trx, GST, MBP, NusA and Halo-tag generally enhance solubility of recombinant proteins. The comparison of expression and solubility of proteins by various fusion tags has been studied previously [40-46]. Although many of these studies represented limited scale examinations, they showed different expression and solubility level by fusion tags and target proteins. Therefore, a protein expression and purification platform would return maximum success by employing a combination of fusion tags.

In this study, we address the following objectives; 1) large-scale protein expression and purification of proteases derived from bacterial pathogens, 2) evaluating the efficiency of different fusion tags using multiple expression vectors in combination, and 3) high throughput assay development for protease screening and confirmation of activities of predicted proteases. A set of 187 protease candidates were selected using bioinformatics tools. These protease candidates were mined from three pathogenic bacteria, *Streptococcus pneumoniae TIGR4*, *Bacillus anthracis Ames *and *Yersinia pestis KIM*. The protease set was used for studies in cloning, expression, purification and function using various fusion tags including hexa-histidine (His-tag), maltose binding protein (MBP), MBP with signal peptide (SP-MBP), disulfide oxydoreductase A (DsbA) and glutathione S-transferase (GST). The combination of the fusion tags provides high success rates (86.6%) in the recovery of soluble expressed target proteins. Nearly all of these (86.1%) were purified successfully. Protease activities of the purified proteins were also examined using a high throughput fluorescence-based assay and zymography, that not only support the annotation as a protease or putative protease but also identified the protease activity of hypothetical proteins and a protein annotated as a non-protease.

## Results

### Overview of the pipeline

**Figure 1:**
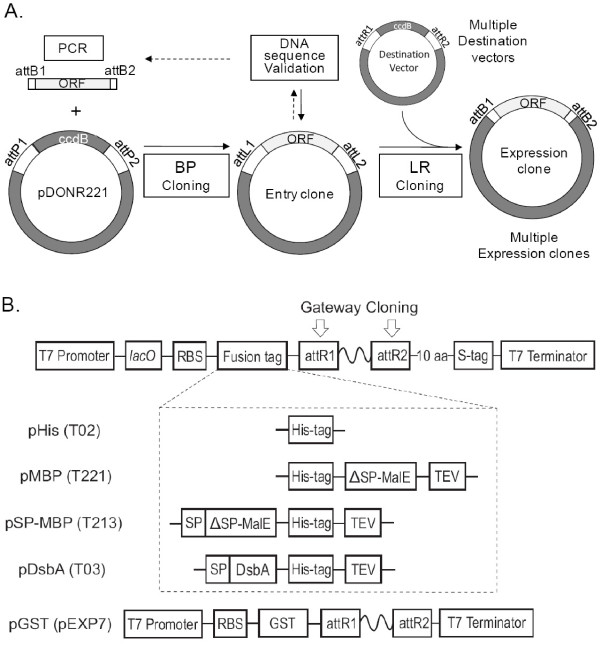
**Gateway compatible expression vectors with different fusion tags**.

**Figure 2:**
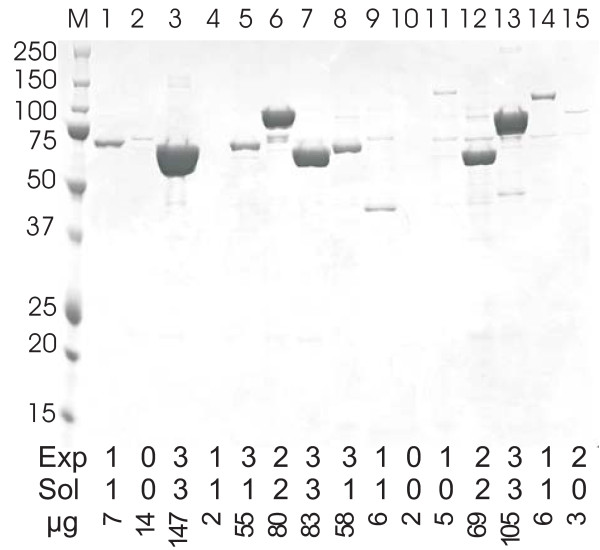
**Coomassie blue stained Nu-PAGE gel of purified proteases**. The proteins were expressed using pMBP vector and purified using Ni-NTA agarose resin as described in Materials and Methods. The purity of proteases was confirmed on the Nu-PAGE gel and the concentrations were determined by Bradford assay with BSA standard curve. (M: marker, 1: SP_1467, conserved hypothetical protein, 2: SP1477, hypothetical protein, 3: y0125, sigma cross-reacting protein 27A, 4: y0137, protease DO, 5: y0720, putative PhnP protein, 6: y0746, cytosol aminopeptidase, 7: y1280, pyrrolidone-carboxylate peptidase, 8: y2013, putative pepetidase, 9: y2057, peptidase U7 family SohB protein, 10: y2527, prolyl oligopeptidase family protein, 11: y2694, conserved hypothetical protein, 12: y3230,4-methyl-5(b-hydroxyethyl)-thiazole monophosphate biosynthesis protein, 13: y3297, proline-specific aminopeptidase, 14: y3855, oligopeptidase A, 15: y3857, putative alkaline metalloproteinase).

The high throughput protein production pipeline is based on a 96-well format procedure. The pipeline consists of cloning, DNA sequence validation, expression, purification, confirmation of protein identity by mass spectroscopy and storage. One of the essential features of our pipeline is the cloning of ORFs into multiple expression vectors. Gateway cloning technology was introduced in the pipeline in order to maintain efficient cloning in this regard (Figure 1). When a single expression vector encoding the His-tag is used to overexpress randomly selected recombinant proteins in *E. coli*, generally, yielded purified, soluble proteins in less than 40% of target genes [29,47]. If the selected genes are toxic to the host cells, the final yields are decreased further. However, when multiple expression vectors are used to prepare protein, the overall success rates of expression, solubility and purification of target proteins can be significantly increased. The Gateway cloning system represents an ideal cloning method to produce multiple expression vectors for use in a high throughput protein production pipeline. Once entry clones are prepared and their sequences are validated, the entry clones may be used to generate expression clones using destination vectors encoding a variety of fusion tags by simply shuttling ORFs into multiple Gateway compatible expression vectors using the recombinase, LR clonase. Multiple expression vectors shown in Figure 1 were constructed based on T7 expression pET system and Gateway cloning system in order to improve expression and solubility of recombinant proteins. Unlike methods using restriction enzymes, Gateway cloning is very efficient and sequence validated entry clones can be used for any Gateway destination vector without further DNA sequence validation. Although the pET system is very powerful, it lacks tight control of expression. The expression control of proteins is critical for successful expression of potentially toxic proteins. Proteases represent a strong example for which expression control is important due to cytotoxic proteolytic activities. The early undesired expression of target proteins using pET expression system was suppressed by addition of glucose in the media and using BL21(DE3)pLysS. A primary carbon source for *E. coli *host, glucose binds to the lac repressor and shut off transcription of the T7 RNA polymerase gene under the control of the lac UV5 promoter. Low level expression of T7 lysozyme from pLysS binds to and inhibits T7 RNA polymerase. Approximately, 86.1% of cloned ORFs were expressed and purified with at least one of expression vectors. By batch purification using 2 mL 96-well filter block, between 2 - 200 μg of recombinant proteins were obtained. Purity of the recombinant proteins was dependent upon the amount of soluble expressed recombinant protein and ranged between 80-95%. The purity of the recombinant proteins were confirmed by Nu-PAGE gel analysis (Figure 2) and the identity of the purified proteins were confirmed by in-gel trypsin digestion followed by MALDI-TOF/TOF analysis.

### Expression vector comparison

The levels of expressions and solubility of recombinant proteins are directly related to the expression vector used and a variety of known and unknown protein characteristics. Consequently, the solubility levels of expressed proteins directly contribute to the final yield of purified proteins as shown in Figure 2. Expression and solubility of the recombinant proteins were estimated by Coomassie blue and/or InVision His-tag staining of Nu-PAGE gels. A simple expression vector, pHis which contains an NH_2_-terminal His-tag, does not enhance solubility of overexpressed proteins [29]. In order to increase solubility of recombinant proteins, additional fusion tags such as MBP, GST or DsbA were used fused at the N-terminus. Two MBP fusion tag expression vectors were constructed. One, named pMBP, lacks the signal peptide (SP), but contains a His-tag just upstream of the MBP segment. The other, named pSP-MBP, was engineered with a SP at the N-terminal end of MBP. Like pSP-MBP, an expression vector, pDsbA also contains a signal peptide as a part of the fusion. In the expression vectors containing a signal peptide, the His-tag is located at the C-terminal end of MBP and DsbA. The expression vector pGST (pEXP7) containing an N-terminal GST fusion tag, lacks a His-tag while all other vectors contain a His-tag for immobilized metal affinity chromatography (IMAC) purification and the GST fused recombinant proteins were purified using Glutathione agarose resin. TEV protease treatment removes tags from recombinant protein for the vectors, pMBP, pSP-MBP and pDsbA.

**Figure 3:**
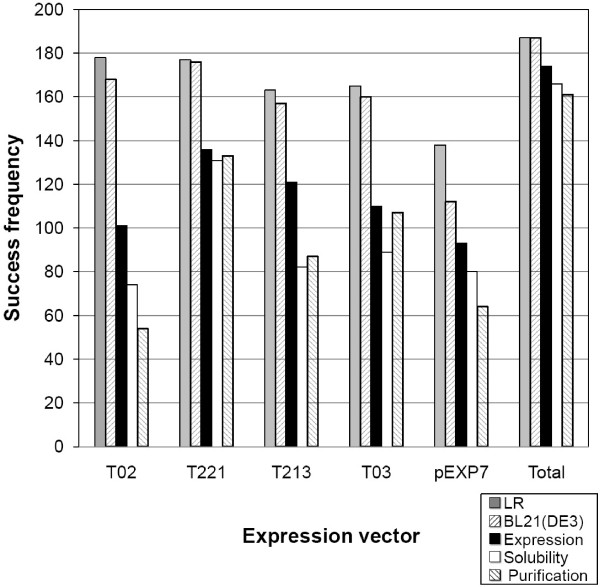
**Comparison of success frequencies of expression vectors at each stage of purification**. Selected entry clones of 187 ORFs in *Bacillus anthracis Ames*, *Streptococcus pneumonia TIGR4 *and *Yersinia pestis KIM*, were used to prepare expression clones using 5 different Gateway comparable vectors, pHis, pMBP, pSP-MBP, pDsbA and pGST. Target proteins were expressed in 96-well blocks by IPTG induction at OD_600 nm _= 0.7-0.9 for 20 hours at 20°C. The harvested cell pellets were resuspended in lysis buffer [50 mM Tris-HCl, 100 mM NaCl, 1 mM DTT at pH 7.8 at 4°C] and lysed using PopCulture and lysonase. The recombinant proteins were purified using Ni-NTA agarose column as described in 'Methods and Materials'.

The expression and solubility of recombinant proteins were examined by Nu-PAGE with His-tag staining (In-Vision) and/or Coomassie blue staining. The expression and solubility of recombinant proteins were scored as "0", "1", "2", and "3" that represent undetectable, low, medium and high expression and solubility, respectively (Additional file 1, **Table S1**). Unexpectedly, not only solubility but also expression success frequency was dependent on the types of fusion tags used as described in Figure 3. The smallest fusion tags in the series, His-tag of pHis expression vector showed the least success frequency for expression and solubility of clones. ORFs cloned into the pSP-MBP or pDsbA containing the *E. coli *signal peptide at the N-terminal end of fusion tags displayed marginally lower success frequencies compared to the other expression vectors examined. The MBP fusion tag in our studies is superior to other tags at all stages from expression to purification. With pMBP alone, 133 proteases (71%) were purified compared to a total 161 different proteases (86.1%) successfully purified by employing a series of expression vectors. The most effective combination of three expression vectors are pSP-MBP, pMBP and pDsbA which resulted in the successful purification of 159 (85%) proteases, nearly the same success as that achieved using all five expression vectors. The best combination of two expression vectors were pMBP and pSP-MBP or pMBP and pDsbA generating success rates of 79.7% and 78.6%, respectively. Expression vectors encoding NH_2_-terminal signal peptide, such as pSP-MBP and pDsbA resulted in a relatively larger number of purified proteins compared to pHis, however to protein recovered from 1 mL culture, resulted in poor yields of purified proteins. In order to increase the final yield of purified proteins, 4 x1 mL cultures were used. The average concentrations of purified proteins from pHis, pMBP, pSP-MBP, pDsbA and pEXP7 were 440, 138, 334, 53 and 21 μg/mL, respectively. The purification success rate of *S. pneumoniae*, *B. anthracis *and *Y. pestis *proteases were 88.2%, 78.0% and 93.4%, respectively. Proteases of *B. anthracis *were marginally more difficult to express and purify.

### Protein localization is a critical factor for protein purification success

**Figure 4:**
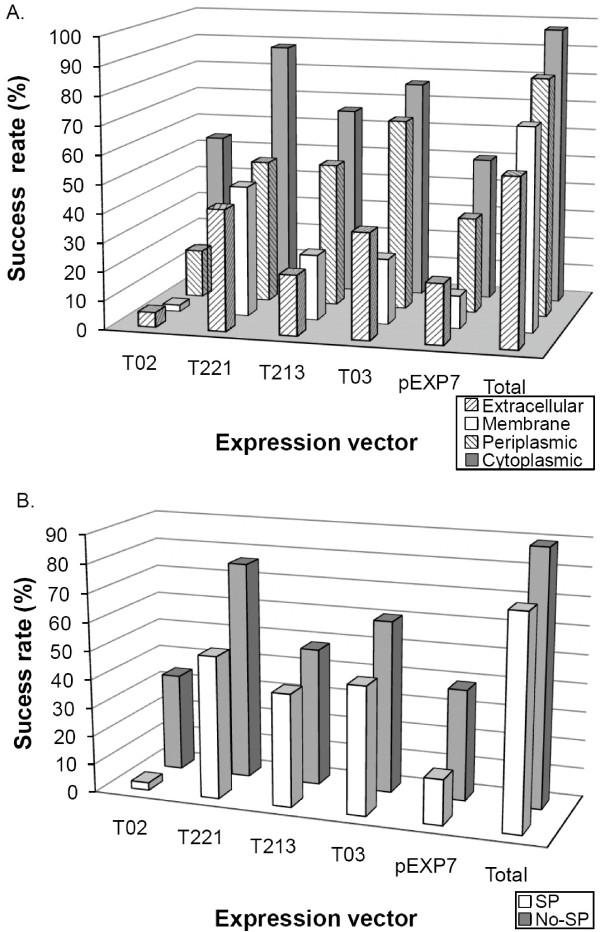
**Dependence of purification success rates on fusion tags and sub-cellular localization of proteins**. Analysis of purification success rate by expression vectors and (A) subcellular localization of proteins or (B) presence of signal peptide.

Characteristics of proteins, such as sub-cellular localization, and localization the presence of signal peptides, are among the most critical factors for expression, solubility and purification. The purified recombinant proteins were confirmed by SDS-PAGE. Because protein purification success rates represent soluble expression of proteins, the correlation between purification and the protein sub-cellular localization were examined. The dependence of purification success rate on the protein localization was clearly evident (Figure 4). Cytoplasmic proteins displayed the highest purification success rate. Proteins with predicted membrane localization and surface were successful in less than 40% of the cases. More than 97% of the attempted cytoplasmic proteins were expressed and purified by at least one of the expression vectors, while 68.1% of non-cytoplasmic proteins were successfully purified. The expression vector, pHis is the least favorable for surface proteins. The other larger fusion proteins increased purification of the surface proteins. The MBP-tag increased the success rate of the surface proteases by approximately 8-fold compared to the His-tag. The presence of signal peptide that targets proteins to the cell surface also decreased the success rate of protein purification. Only one of 38 attempted proteins containing native signal peptide were purified using the pHis expression vector. The expression vector with the GST-tag also performed poorly for proteins containing signal peptide. Half of the proteins containing signal peptide were purified using an expression vector, pMBP, which was also the most successful vector for both the presence and the absence of signal peptide. Approximately 20-fold more proteases containing signal peptide were purified with pMBP compared to pHis. With a combination of 5 expression vectors, 79% of proteins containing native signal peptide were purified.

### Protease activity characterization

**Figure 5:**
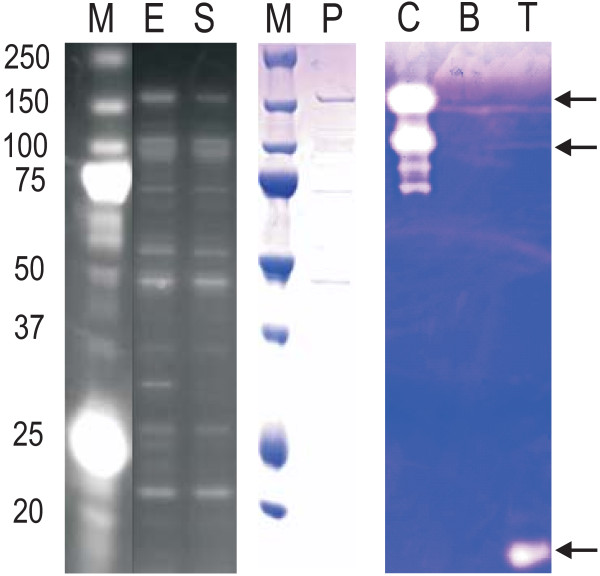
**Detection of protease activity using gelatin zymogram**. A putative collagenase in *Bacillus anthracis Ames *was cloned in pMBP and expressed in BL21(DE3)pLysS. Expression (E) and solubility (S) of the putative collagenase (BA0555) were confirmed by His-vision staining on Nu-PAGE. The purified protein was run on Nu-PAGE gel and stained with Coomassie blue to confirm the purity (P). Gelatin zymogram was performed to examine protease activity using 2 ng of the purified putative collagenase (C). Purification background using *E. coli *supernatant (B) and 2.5 ng of trypsin (T) were used as a negative and a positive control, respectively.

The activities of purified proteases were examined using gelatin zymography and/or a fluorescence-based assay. Although zymography has limitations associated with the case of variability of proteins refolding to a native form after separation on SDS-PAGE, it is still a robust method for confirming protease activities. An example of a zymogram is shown in Figure 5. A putative microbial collagenase of *B. anthracis *was applied to zymographic analysis. The expression of the collagenase was very low and roughly 50% of the protein was solubilized. The total amount of the purified putative collagenase from 1 mL culture was only approximately 1.6 μg. The enzyme activity was confirmed by applying 2 ng of the collagenase on gelatin zymography. The result was shown in the lane C in Figure 5. The activity of the collagenase was compared with the activity of 2.5 ng of Trypsin shown in lane T. Three putative microbial collagenases in *B. anthracis *were annotated and all of them were purified with a series of fusion tags. All of the purified collagenases showed activities for gelatin but not for BZAR (Table 1 Additional file 1**Table S1**). In addition to the putative collagenases, activities of four more proteases were confirmed by zymography.

**Table 1:** Activity screen of purified putative microbial collagenases in *Bacillus anthracis Ames *using different expression vectors

Gene	pHis (T02)		pMBP (T221)		pSP-MBP (T213)		pDsbA (T03)		pGST (pEXP7)	
	
	DQ	Gelatin	DQ	Gelatin	DQ	Gelatin	DQ	Gelatin	DQ	Gelatin
BA0555	N/A	N/A	+	+	+	+	+	+	N/A	N/A
BA3299	N/A	N/A	+	+	+	+	N/A	N/A	-	+
BA3584	+	+	+	+	+	+	+	+	-	+

N/A: not available, +: activity detected and -: no activity detected

**Figure 6:**
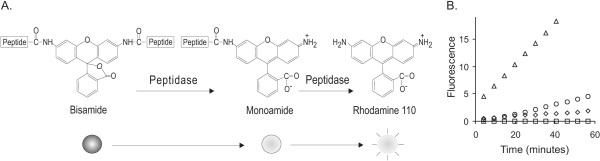
**Protease activity screen using a fluorescence substrate, BZAR**. A. The BZAR (Rhodamine 110, bis-(N-CBZ-L-arginine amide), dihydrochloride) is a general substrate of serine proteases. Upon protease cleavage, the nonfluorescent bisamide derivative of rhodamine 110 is first converted to the fluorescent monoamide and then to rhodamine 110, with a further increase in fluorescence. B. Time course measurements of protease cleavage of BZAR. The protease cleavage reactions were montiored by time fluorescence measurements (λ_ex_: 485 nm, λ_em_: 535 nm) using a GENeo Pro (Tecan). The increase of fluorescence represents protease cleavage reactions. Δ: y3353, putative kinase, ○: trypsin, ◊: y1992: putative carboxypeptidase, and □: SP1780, oligoendopeptidase.

Alternative fluorescence assay for serine proteases were performed using a well-known Rhodamine 110-based serine protease substrate, bis-(CBZ-L-arginine amide), dihydrochloride (BZAR). The structure of BZAR and scheme are shown in Figure 6. Upon enzymatic cleavage, the non-fluorescent bisamide substrate in converted to monoamide and then to rhodamin110 which can be easily monitored by fluorescence increase. The fluorescence changes of BZAR upon adding proteases were monitor for one hour. Positive slopes represent the hydrolysis of peptide in BZAR. Two criteria were used to confirm the protease activity, the slope and the ratio of signal to noise (S/N). Thresholds of the slope and ratio of S/N were defined as 0.015 and 2, respectively. A total of 74 proteases were confirmed to have activity by fluorescence assays using BZAR and DQ and gelatin zymography (Table 2). Among the list of confirmed proteases were 44 hypothetical proteins which were predicted to be proteases based on analysis using Prosite motif search, protein family analysis (PFAM) and/or, Clusters of Orthologous Groups of proteins (COGs), and 43.2% of the candidates tested displayed protease activity. A protein annotated as a putative kinase in *Y.pestis *also displayed protease activity.

**Table 2:** Summary of protease activity identification

	Total purified proteases	Activity confirmed	Hypothetical proteins	Activity confirmed
Total	161	74 (46.0%)	44	19 (43.2%)

The protease activity was detected by either fluorescence assay using BZAR or gelatin zymography.

Confirmed activities of purified proteases were dependent on the fusion tags used. A relatively large number of proteases were purified using the pMBP expression vector, but only approximately 11% of them were confirmed for protease activity. A similar numbers of proteins were confirmed that were purified from other vectors for protease activity. Nearly 60% of purified proteins using pSP-MBP displayed protease activity. The pSP-MBP vector contains maltose binding protein (MBP) as an N-terminal fusion tag like pMBP, and the *E. coli *signal peptide at the N-terminus of the MBP and hexa-histidine tag between the MBP and Gateway cloning site. Based on mass spectroscopy analysis, the signal peptides were processed during expression of the target proteins (Data not shown). Although purification yields of proteases using the expression vector, pSP-MBP were low, the frequencies of active proteases were much higher than protease with other fusion tags.

## Discussion

In this study, two elements of the previous platform of protein expression and purification have been modified - *E. coli *expression strain and multiple fusion tags [29,40-46]. All of the vectors contain T7 promoters for overexpression in the same *E. coli *expression strain, BL21(DE3)pLysS in order to maintain tight control of protein expression while BLR(DE3) was used for large-scale expression and purification of proteins in *Streptococcus pneumoniae TIGR4 *in a previous study [29,48]. The modified platform used IPTG induction while the previous platform was based on a combination of autoinduction and IPTG induction. We were able to successfully express only 15% of the proteases as soluble protein with T02 (pHis) vector in the previous study. One of the major hurdles was the preparation of expression clones and transformation into BLR(DE3) as well as the expression soluble proteins. For the same targets in this study, previously 26% of them were expressed in soluble form in BLR(DE3) while 38% of them were expressed in soluble form in BL21(DE3)pLysS.

In order to efficiently clone target genes into five expression vectors, a Gateway based cloning strategy was employed in a high throughput protein expression and purification pipeline [29,47,48]. The prepared entry clones of target genes were sequence validated and used to shuttle the cloned ORFs into Gateway compatible expression vectors. The cloning efficiency of each expression vector was high except in the case of pEXP7. The extra amino acids residues present in recombinant proteins due to Gateway cloning site attB1 and attB2 were identical in all of the expressed proteins.

Therefore, the expression, solubility and purification yields were mostly dependent on the specific fusion tag used. The His-tag is one of the most popular fusion partners for IMAC, but it showed lower success in expression compared to other fusion tags. In contrast, the 44.3 KDa signal peptide deleted maltose binding protein (MBP) improved the expression and solubility of target proteins substantially [40,41,49]. The frequency and level of expression achieved with MBP is superior to other fusion tags examined in this study. Almost all expressed proteins with the MBP tag were soluble. However, expressed proteins containing a signal peptide at the N-terminus of MBP (SP-MBP) showed different patterns of expression and solubility compared to the proteins with MBP fusion alone. Two expression vectors, pSP-MBP and pDsbA contain signal peptides at the N-terminal end of the fusion tags and the frequencies of expression and soluble proteins expressed using two vectors were similar. DsbA, an *E. coli *periplasmic enzyme also has signal peptide to allow protein translocation across the membrane and the catalysis of disulfide bond formation [32]. When recombinant proteins targeted to the periplasm are overexpressed, the translocation machinery of the *E. coli *host cell may become overloaded leading to a decrease in the translocation efficiency. The recombinant proteins may become trapped in the inner membrane; accumulate as inclusion bodies, or may be proteolyzed in the cytoplasm [50-52].

According to the mass spectroscopy analysis of purified recombinant proteins, the signal peptides of the fusion tags SP-MBP and DsbA are effectively removed from the N-terminal end of expressed proteins. The signal peptide of the protein of interest itself was not processed due to the location of SP between MBP tag and protein of interest. The low yield purification of SP-MBP tagged proteins were overcome by scaling up the volume of bacterial cultures by four-fold. However, the presence of the signal peptide associated with the protein of interest did not correlate to functional activity of the protease expressed with SP-MBP.

Based on the protease assays performed, 19 proteins annotated as hypothetical proteins were confirmed to have protease activity. One of the proteins, annotated as a putative kinase, showed protease activity based on the BZAR assay. The protein is annotated as a putative kinase (YPO0966) in *Yersinia pestis *CO92 and the orthologous protein in *Yersinia pestis *KIM is annotated as a hypothetical protein (y3353). According to NCBI Blast, it belong to Pfam01551 (protein family) which encodes peptidase M23. This protein is also a member of COG0739 (Clusters of Orthologous Groups of proteins) of membrane proteins related to metallo-endopeptidases. The three putative collagenases in *Bacillus anthracis*, BA0555, BA3299 and BA3584 were also confirmed in their putative activity by gelatin zymography and a robust DQ gelatin fluorescence assay. The activities of the putative collagenases clearly were observed by both methods except in the case of GST-tagged putative collagenases. The activities of the purified GST-tagged putative collagenases would be recovered during denaturation and renaturation process during zymography. According to alignment using LALIGN http://www.ch.embnet.org/software/LALIGN_form.html, pair-wise identity scores of the three putative collagenases are between 58.2% - 71.6% [53]. All of them contain a peptidase M9 domain which is found in microbial collagenase metalloproteases. These results support the importance of experimental validation of bioinformatically annotated functional predictions.

Fusion tags are one of several significant components for successful expression and solubility levels of proteins [54]. The most appropriate choice of fusion tags for soluble expression of proteins is varied from target to target. Therefore, a combination of fusion tags may result in the highest recovery of soluble proteins. The presence of recombinant signal peptide is also critical for soluble expression and the maintenance of protein activity [55]. Not only endogenous but also recombinant signal sequences may be used by the translocation machinery. It has been reported that codon optimized signal peptides of eukaryotic origin were efficiently translocated as recombinant protein into periplasm of *E. coli *[56]. In the current study, we used a generic *E. coli *signal peptide fused at the N-terminus to the maltose binding protein since the native signal peptides encoded by target proteins may not support proper translocation of the target proteins. The native signal peptides of target proteins can be used when using a C-terminal fusion-tag system. Using the pSP-MBP vector, 46.5% target proteins were purified and the majority displayed protease activity. By contrast, 71.1% of the targets were purified and only 11.3% displayed protease activity when using pMBP. These results suggest that proper translocation of some proteins into the *E. coli *periplasm, where formation and isomerization of disulfide bonds occurs, is significant for maintenance of protein activity and stability [55,57]. The reduced purification success observed when using pSP-MBP was due to low protein solubility perhaps related to inefficient translocation to the periplasmic space. It is possible that, the solubility of SP-MBP tagged recombinant proteins could be improved by expressing proteins in an *E. coli *strain engineered to overexpress the periplasimc machinery [52]. Alternatively, disulfide bond formation of target proteins may be promoted in the cytoplasm using thioredoxin reductase deficient *E. coli *strains such as BL21trx(DE3) [57-61].

Membrane proteins are very challenging targets. We purified approximately 70% of the predicted membrane proteins using the combination of 5 different fusion tags. The majority of the purified membrane proteins contain six or fewer transmembrane segments. The correlation between the number of transmembrane segments in target proteins and purification success was previously reported [62]. Only one membrane protein was successfully purified using the His-tag, whereas the large fusion tags, MBP, SP-MBP and DsbA enhanced solubility of expressed membrane proteins resulting in 45.5%, 22.7% and 22.7% of the targets being successfully purified, respectively. The combination of these large fusion tags increases purification success rate of membrane proteins. An alternative high throughput method for membrane proteins encoding more than six transmembrane segments is the cell free expression strategy using liposomes or detergent [63,64].

## Conclusions

We modified a previously defined high throughput protein production and successfully adapted it for cloning, expression and purification of a set of 187 proteases using multiple Gateway compatible expression vectors. The 96-well, 1 mL cell culture platform yields enough protein for the combinatorial functional analyses using high throughput fluorescence assays and zymography to identify protease activity of the purified proteins. This high throughput pipeline was successfully used for experimental confirmation of gene annotation and bioinformatics functional predictions, and can be easily adapted for both initial screening of protein expression and solubility for scale-up production for functional and/or structural studies.

## Methods

### Construction of Gateway Compatible pET-destination vectors

Construction of the *pET-Dest-TIGR02 (abbreviation is T02 or pHis) *vector was previously described in Kwon et al [29]. In addition to the T02, pET-Dest-TIGR221(T221 or pMBP), *pET-Dest-TIGR213 (T213 or pSP-MBP) and pET-Dest-TIGR03 (T03 or pDsbA) *were constructed. The signal sequence deleted version of the *malE *gene of pMAL-c2x (New England Biolabs, Ipswich, MA) was used to construct T221. The malE gene was amplified using three oligonucleotides in a 50 μL reaction with PCR High Fidelity Supermix (Invitrogen): 1) 20 pmoles PX006 for 5' end of *malE*, 2) 1 pmole PX007.2 for 3' end of *malE*, and 3) 20 pmoles PX0041 for addition of DNA sequence encoding the TEV protease cleavage site at the carboxyl-terminus of maltose binding protein (Table 3). The resulting products were purified using GFX DNA and Gel Band Purification Kit (GE Healthcare, Piscataway, NJ) and digested with PmlI. T02 vector was digested with PmlI followed by treatment with CIP. The *malE*-TEV fragment was ligated into the T02 vector using the Rapid Ligase (Roche, Basel, Switzerland). DB3.1 [F^- ^*gyr*A462 *endA*1 Δ*sr*1-*rec*A) *mcr*B *mrr hsd*S20(r_B_^-^, m_B_^-^) *sup*E44 *ara*14 *gal*K2 *lac*Y1 *pro*A2 *rps*L20(Sm^r^) *xyl5 *Δ*leu mtl*1] cells (Invitrogen, Carlsbad, CA) were chemically transformed and transformants were plated on LB agar containing 100 μg/mL ampicillin and 34 μg/mL chloramphenicol. Proper orientation of the cassette was screened by PCR and the vector was validated by DNA sequencing. Two expression vectors, pET-Dest-TIGR213 (T213 or pSP-MBP) and pET-Dest-TIGR03 (T03 or pDsbA), containing signal peptide were constructed. With signal peptide The full *malE *gene of pMAL-p2x (New England Biolabs) was amplified using 20 pmoles PX020, 1 pmole PX021, and 20 pmoles PX029 in a 50 μL reaction with PCR High Fidelity Supermix (Invitrogen) to construct T213. The full *dsbA *gene of pET39b+ (EMD Biosciences) was amplified using 20 pmoles PX023, 1 pmole PX024, and 20 pmoles PX029 to construct T03. The sequences of oligonucleotides are shown in Table 3. The resulting products were purified as described above and digested with NcoI and KpnI. The pET45b+ (EMD Biosciences, San Diego, CA) was digested with NcoI and KpnI followed by treatment with CIP. The full *malE*-His-tag-TEV and *dsbA*-His-tag-TEV fragments were ligated to the pET45b+ backbone to create the pSP-MBP-His-tag intermediate vector and pDsbA-His-tag, respectively. The addition of the Gateway cassette rfc.1 to these intermediate vectors parallels the T02.

**Table 3:** Oligonucleotide sequences used for construction of expression vectors

Name	Oligonucleotide sequence
PX006	GGGGCACGTGGGTATGAAAATCGAAGAAGGTAAACTG
PX007.2	CTGGAAGTACAGGTTCTCACCGCTCGAATTAGTCTGCGCGTC
PX0041	GGGGCACGTGACCACCCTGGAAGTACAGGTCTC
PX0020	GGGGCCATGGCAATGAAAATAAAAACAGGTGCACGCA
PX0021	GTGATGGTGGTGGTGATGACCCGAACCGCTCGAGCTCGAATTAGTCTGCGC
PX0023	GGGGCCATGGCTATGAAAAAGATTTGGCTGGCGCTGG
PX0024	GTGATGGTGGTGGTGATGACCCGAACCGCTAGTTGATCCTTTTTTCTCGCTTAAGTATTTCAC
PX0029	GGGGGGTACCACCACCCTGGAAGTACAGGTTCTCACCGGCCGAGTGATGGTGGTGGTGAT

### Selection and prediction of proteases

Targets of putative proteases in *Bacillus anthracis Ames*, *Streptococcuss pneumoniae TIGR4 *and *Yersinia pestis Kim *were selected based on annotation of ORFs in J. Craig Venter Institute Comprehensive Microbial Resource (CMR) http://cmr.jcvi.org/tigr-scripts/CMR/CmrHomePage.cgi, PROSITE search http://au.expasy.org/tools/scanprosite/), TIGRFAM/PFAM search http://blast.jcvi.org/web-hmm/. Presence of signal peptide was done using SignalP http://www.cbs.dtu.dk/services/SignalP/. Cellular localization of the target proteins were predicted using PSORT-B http://www.psort.org/psortb/[65].

### Cloning, expression and purification

Entry clones used to construct expression clones were obtained from the PFGRC http://pfgrc.jcvi.org/. The pathogen entry clone sets are available from the Biodefense Emerging Infections Research Resources Repository http://www.beiresources.org/: *Bacillus anthracis Ames *(NR-19272), *Streptococcus pneumoniae TIGR4 *(NR-19278), and *Yersinia pestis KIM *(NR-19280). Expression clones were generated by following the procedures as described in Kwon *et al. *[29]. The cloned inserts were verified by DNA sequencing. The destination clones were transformed into BL21(DE3)pLysS competent cells which were prepared using the Z-competent *E. coli *transformation kit (Zymo Research, Orange, CA), and frozen cultures were prepared as described in Kwon *et al. *[29].

Recombinant proteins were overexpressed in BL21(DE3)pLysS using five expression vectors, T02 (pHis), T221 (pMBP), T213 (pSP-MBP), T213(pDsbA) and pEXP7 (pGST). The cells were grown in 96-well 2 mL blocks (Whatman Inc., Piscataway, NJ) containing 1 mL 2xYT medium, 100 μg/mL ampicillin and 34 μg/mL chloramphenicol. The *E. coli *cells were incubated at 37°C in a Multitron incubator shaker (INFORS HT, Bottmingen, Switzerland). When cells achieved an OD_600 nm _between 0.7 and 0.9, 1 mM final concentration of isopropyl β-D-1-thiogalactopyranoside (IPTG) was added to the cultures and incubated for an additional 18 to 20 hours at 25°C. Cells were harvested by centrifugation at 2000 × g for 20 minutes, and the cell pellets were stored at -20°C.

The recombinant proteins containing the His-tag were purified using Ni-NTA agarose resin. Cell pellets were resuspended in 0.4 mL low salt lysis buffer [50 mM Tris-HCl, 100 mM NaCl, 5 mM imidazole, 1 mM DTT, pH 8.0 at 4°C] with 1.2 μL Lysonase Bioprocess (EMD Biosciences). Each cell suspension was incubated on ice for 20 minutes and 65 μL PopCulture (EMD Bioscience) was added to lyse the cells completely. The lysates were incubated on a microtiter plate shaker at 4°C for 30 minutes and the NaCl concentration was adjusted to 300 mM. Soluble fractions were separated by centrifugation at 2400 × g at 4°C for 1 hour in a bench top centrifuge (Eppendorf, 5810R). The supernatants were applied to 96-well filter blocks containing 100 μL Ni-NTA agarose resin (50% v/v slurry) in a Whatman 2 mL Unifilter block with GF/C filter (Whatman Inc.). The block was sealed with a capmat and a parafilm-covered v-bottom 96-well plate and rotated on the tube rotator for 1 hour in the cold room to bind the protein to resin. The resins were collected by quick centrifugation and buffer was removed using an automated liquid handler, Biomek FX. The parafilm-covered 96-well plate was removed from the filter block and the filter block was placed on a new deep-well block. The resin was washed with 500 μL wash buffer [50 mM Tris-HCl, 10 mM imidazole, 300 mM NaCl, 1 mM DTT, 0.1% Triton X-100, pH 8.0 at 4°C] 5 times. Recombinant proteins were eluted in 150 μL [50 mM Tris-HCl, 300 mM NaCl, 200 mM imidazole, 1 mM DTT, pH 8.0 at 4°C] twice, and imidazole was removed by exchange into storage buffer [50 mM Tris-HCl, 300 mM NaCl, 5% glycerol, 1 mM DTT, pH 7.5 at 4°C] using an Ultracel-10 96-well filtration devices (Millipore, Billerica, MA). Purity levels of the resulting proteins were confirmed on Nu-PAGE gels (Invitrogen, Carlsbad, CA). Recombinant proteins visualized by Nu-PAGE were subjected to in-gel digestion with trypsin, and their identities were determined by MALDI-TOF/TOF analysis as previously described [66]. The purified protein concentrations were determined by absorbance at 280 nm using a GENios Pro plate reader (Tecan). The extinction coefficient of each recombinant protein was calculated from amino acid sequence of the fusion tag and the target protein [67]. Purified proteins were stored at -80°C. The GST-tagged proteins were purified by following the same procedure for His-tagged protein purification using glutathione agarose resin and GST elution buffer [50 mM Tris-HCl, pH 8.0 at 4°C, 1% reduced glutathione].

### Zymography

Zymography was performed with 10% Tris-Glycine gels containing 0.1% gelatin (Invitrogen) as a substrate. Initially, the protease samples in the range of 10 ng to 5 μg were denatured with SDS sample buffer and subjected to electrophoresis at room temperature. After electrophoresis, gels were removed and incubated in Zymogram Renaturing Buffer (Invitrogen) for 30 minutes at room temperature with gentle agitation to renature the separated proteases by replacing SDS. The gels were then equilibrated in Zymogram Developing Buffer containing divalent metal cation suitable for enzymatic activity (Invitrogen) for 30 minutes at room temperature with gentle agitation. After decanting the buffer, fresh 1× Zymogram Developing Buffer was added to the gels. The gels were then at 37°C overnight for maximum sensitivity. The protease activity was visualized by staining with Coomassie Brilliant Blue R250 and destaining thoroughly in destaining solution [5% methanol, 7.5% acetic acid]. Areas of digestion appearing clear against a dark blue background represent enzymatic activity.

### Fluorescence measurements

Fluorescence substrates, BZAR (rhodamine 110, bis-(CBZ-L-arginine amide), dihydrochloride) and DQ gelatin were used to detect protease activity of purified proteins. The reaction with BZAR was initiated by adding 5 μL purified protein stock into 45 μL 30 nM substrate prepared in reaction buffer [10 mM Tris pH 7.5 at 25°C, 100 μg/mL BSA] in a half-size 96-well plate. Time course fluorescence measurements of the reactions were made at λ_ex _= 485 nm and λ_em _= 535 nm using GENios Pro (Tecan). In each well of a 96-well plate, 45 μL 12.5 μg/mL DQ gelatin was prepared in DQ-gelatin reaction buffer [50 mM Tris-HCl, 150 mM NaCl, 5 mM CaCl_2_, pH 7.6] and proteolysis reaction was performed by adding 5 μL purified proteins. The reactions were incubated at 25°C and time course fluorescence measurements were performed at the same setting as BZAR assays.

## Authors' contributions

KK, designed experiments, developed protein expression and purification pipeline and assays, interpreted the data, wrote the manuscript; JH; performed experiments, managed the pipeline and reviewed the manuscript; CG, constructed expression vectors and performed experiments; SL and YD, performed experiments; RP, participated in the study design, manuscript development and its review; RDF, participated in the design of the study; SNP, participated in the design of the study and the review of the manuscript.

## Supplementary Material

Additional file 1 **Table S1 - Data table of protease preparation in five expression vectors and protease activity assay**. The selected proteases of *Streptococcus pneumoniae*, *Bacillus anthracis *and *Yersinia pestis *were cloned into five expression vectors, pHis (T02), pMBP (T221), pSP-MBP (T213), pDsbA (T03) and pGST (pEXP7). Data from cloning to protease assay are presented for expression vector. "LR" column represents LR cloning of target ORFs from entry clone. Scores "1" and "0" in "LR" column represent "success" and "fail", respectively. Transformation column represents the number of colonies after transformation into BL21(DE3)pLysS. The scores, "0", "1", "2", and "3" in the columns of 'Expression' and 'Solubility' represent none, low, medium and high expression and solubility, respectively. "[Purified] μg/mL" is the concentration of purified recombinant protein and "Purified (PAGE)" for pGST (pEXP7) represents success ("1") or fail ("0") in purification. "BZAR (S/N)" is the ratio of fluorescence in the presence to the absence of protease after 1 hour incubation. "BZAR (slope)" is the rate of fluorescence change in the presence of a given protease. Combining S/N and slope, protease activity to BZAR was determined and the result is shown in column of "BZAR". The column of "Gelatin" is the result of gelatin zymography, and the numbers represent relative activity-1: weak. 2: medium and 3: strong. "DQ (slope)" is the rate of fluorescence change in the presence of a given protease. "Family" column described peptidase family grouped, and the first letter represents catalytic type: A, aspartic; C, cystein,; M, metallic; S, serine; and U, unknown. Click here for file

## References

[B1] Gosalia DN Salisbury CM Ellman JA Diamond SL High throughput substrate specificity profiling of serine and cysteine proteases using solutionphase fluorogenic peptide microarrays Mol Cell Proteomics 2005 4 5 626 636 10.1074/mcp.M500004-MCP200 15705970

[B2] Rawlings ND O'Brien E Barrett AJ MEROPS: the protease database Nucleic Acids Res 2002 30 1 343 346 10.1093/nar/30.1.343 11752332 PMC99100

[B3] Soh UJ Dores MR Chen B Trejo J Signal transduction by protease-activated receptors Br J Pharmacol 2010 160 2 191 203 10.1111/j.1476-5381.2010.00705.x 20423334 PMC2874842

[B4] Burrows JF Kelvin AA McFarlane C Burden RE McGrattan MJ De la Vega M Govender U Quinn DJ Dib K Gadina M USP17 regulates Ras activation and cell proliferation by blocking RCE1 activity J Biol Chem 2009 284 14 9587 9595 10.1074/jbc.M807216200 19188362 PMC2666611

[B5] Kumar S Caspase function in programmed cell death Cell Death Differ 2007 14 1 32 43 10.1038/sj.cdd.4402060 17082813

[B6] Huston WM Bacterial proteases from the intracellular vacuole niche; protease conservation and adaptation for pathogenic advantage FEMS Immunol Med Microbiol 2010 59 1 1 10 10.1111/j.1574-695X.2010.00672.x 20402770

[B7] Ades SE Proteolysis: Adaptor, adaptor, catch me a catch Curr Biol 2004 14 21 R924 926 10.1016/j.cub.2004.10.015 15530384

[B8] Bolon DN Grant RA Baker TA Sauer RT Nucleotide-dependent substrate handoff from the SspB adaptor to the AAA+ ClpXP protease Mol Cell 2004 16 3 343 350 10.1016/j.molcel.2004.10.001 15525508

[B9] Orth K Xu Z Mudgett MB Bao ZQ Palmer LE Bliska JB Mangel WF Staskawicz B Dixon JE Disruption of signaling by Yersinia effector YopJ, a ubiquitin-like protein protease Science 2000 290 5496 1594 1597 11090361 10.1126/science.290.5496.1594

[B10] Jackson MW Silva-Herzog E Plano GV The ATP-dependent ClpXP and Lon proteases regulate expression of the Yersinia pestis type III secretion system via regulated proteolysis of YmoA, a small histone-like protein Mol Microbiol 2004 54 5 1364 1378 10.1111/j.1365-2958.2004.04353.x 15554975

[B11] Bergo MO Lieu HD Gavino BJ Ambroziak P Otto JC Casey PJ Walker QM Young SG On the physiological importance of endoproteolysis of CAAX proteins: heart-specific RCE1 knockout mice develop a lethal cardiomyopathy J Biol Chem 2004 279 6 4729 4736 14625273 10.1074/jbc.M310081200

[B12] Schneider DR Sigel SP Parker CD Characterization of Vibrio cholerae protease activities with peptide digest analysis J Clin Microbiol 1981 13 1 80 84 6161944 10.1128/jcm.13.1.80-84.1981 PMC273726

[B13] Malloy JL Veldhuizen RA Thibodeaux BA O'Callaghan RJ Wright JR Pseudomonas aeruginosa protease IV degrades surfactant proteins and inhibits surfactant host defense and biophysical functions Am J Physiol Lung Cell Mol Physiol 2005 288 2 L409 418 15516485 10.1152/ajplung.00322.2004

[B14] O'Brien-Simpson NM Pathirana RD Paolini RA Chen YY Veith PD Tam V Ally N Pike RN Reynolds EC An immune response directed to proteinase and adhesin functional epitopes protects against Porphyromonas gingivalis-induced periodontal bone loss J Immunol 2005 175 6 3980 3989 16148146 10.4049/jimmunol.175.6.3980

[B15] Engel LS Hill JM Moreau JM Green LC Hobden JA O'Callaghan RJ Pseudomonas aeruginosa protease IV produces corneal damage and contributes to bacterial virulence Invest Ophthalmol Vis Sci 1998 39 3 662 665 9501882

[B16] Shao F Merritt PM Bao Z Innes RW Dixon JE A Yersinia effector and a Pseudomonas avirulence protein define a family of cysteine proteases functioning in bacterial pathogenesis Cell 2002 109 5 575 588 10.1016/S0092-8674(02)00766-3 12062101

[B17] Hava DL Camilli A Large-scale identification of serotype 4 Streptococcus pneumoniae virulence factors Mol Microbiol 2002 45 5 1389 1406 12207705 PMC2788772

[B18] Abbenante G Fairlie DP Protease inhibitors in the clinic Med Chem 2005 1 1 71 104 10.2174/1573406053402569 16789888

[B19] Tsuji N Miyoshi T Battsetseg B Matsuo T Xuan X Fujisaki K A cysteine protease is critical for Babesia spp. transmission in Haemaphysalis ticks PLoS Pathog 2008 4 5 e1000062 10.1371/journal.ppat.1000062 18483546 PMC2358973

[B20] Tomlinson SM Malmstrom RD Russo A Mueller N Pang YP Watowich SJ Structure-based discovery of dengue virus protease inhibitors Antiviral Res 2009 82 3 110 114 10.1016/j.antiviral.2009.02.190 19428601 PMC2680748

[B21] Polgâar L Mechanisms of protease action 1989 Boca Raton, Fla.: CRC Press

[B22] Carter P Wells JA Dissecting the catalytic triad of a serine protease Nature 1988 332 6164 564 568 10.1038/332564a0 3282170

[B23] Lewis AP Thomas PJ A novel clan of zinc metallopeptidases with possible intramembrane cleavage properties Protein Sci 1999 8 2 439 442 10048339 10.1110/ps.8.2.439 PMC2144267

[B24] Hooper NM Families of zinc metalloproteases FEBS Lett 1994 354 1 1 6 10.1016/0014-5793(94)01079-X 7957888

[B25] Rawlings ND Barrett AJ Bateman A MEROPS: the peptidase database Nucleic Acids Res 2010 38 Database D227 233 10.1093/nar/gkp971 PMC2808883 19892822

[B26] Tang XM Shen W Lakay FM Shao WL Wang ZX Prior BA Zhuge J Cloning and over-expression of an alkaline protease from Bacillus licheniformis Biotechnol Lett 2004 26 12 975 979 15269522 10.1023/b:bile.0000030042.91094.38

[B27] Komai T Ishikawa Y Yagi R Suzuki-Sunagawa H Nishigaki T Handa H Development of HIV-1 protease expression methods using the T7 phage promoter system Appl Microbiol Biotechnol 1997 47 3 241 245 10.1007/s002530050920 9114515

[B28] Li AN Li DC Cloning, expression and characterization of the serine protease gene from Chaetomium thermophilum J Appl Microbiol 2009 106 2 369 380 10.1111/j.1365-2672.2008.04042.x 19200305

[B29] Kwon K Pieper R Shallom S Grose C Kwon E Do Y Latham S Alami H Huang ST Gatlin C A correlation analysis of protein characteristics associated with genome-wide high throughput expression and solubility of Streptococcus pneumoniae proteins Protein Expr Purif 2007 55 2 368 378 10.1016/j.pep.2007.06.006 17703947

[B30] Nilsson J Stahl S Lundeberg J Uhlen M Nygren PA Affinity fusion strategies for detection, purification, and immobilization of recombinant proteins Protein Expr Purif 1997 11 1 1 16 10.1006/prep.1997.0767 9325133

[B31] Lesley SA High-throughput proteomics: protein expression and purification in the postgenomic world Protein Expr Purif 2001 22 2 159 164 10.1006/prep.2001.1465 11437590

[B32] Terpe K Overview of tag protein fusions: from molecular and biochemical fundamentals to commercial systems Appl Microbiol Biotechnol 2003 60 5 523 533 12536251 10.1007/s00253-002-1158-6

[B33] Braun P LaBaer J High throughput protein production for functional proteomics Trends Biotechnol 2003 21 9 383 388 10.1016/S0167-7799(03)00189-6 12948670

[B34] Esposito D Chatterjee DK Enhancement of soluble protein expression through the use of fusion tags Curr Opin Biotechnol 2006 17 4 353 358 10.1016/j.copbio.2006.06.003 16781139

[B35] Stevens RC Design of high-throughput methods of protein production for structural biology Structure 2000 8 9 R177 185 10.1016/S0969-2126(00)00193-3 10986469

[B36] Sugar FJ Jenney FE Poole FL Brereton PS Izumi M Shah C Adams MW Comparison of small- and large-scale expression of selected Pyrococcus furiosus genes as an aid to high-throughput protein production J Struct Funct Genomics 2005 6 2-3 149 158 10.1007/s10969-005-3341-3 16211512

[B37] Hack NJ Billups B Guthrie PB Rogers JH Muir EM Parks TN Kater SB Green fluorescent protein as a quantitative tool J Neurosci Methods 2000 95 2 177 184 10.1016/S0165-0270(99)00178-8 10752489

[B38] Kim JS Raines RT Ribonuclease S-peptide as a carrier in fusion proteins Protein Sci 1993 2 3 348 356 8453373 10.1002/pro.5560020307 PMC2142386

[B39] Los GV Encell LP McDougall MG Hartzell DD Karassina N Zimprich C Wood MG Learish R Ohana RF Urh M HaloTag: a novel protein labeling technology for cell imaging and protein analysis ACS Chem Biol 2008 3 6 373 382 10.1021/cb800025k 18533659

[B40] Hammarstrom M Hellgren N van Den Berg S Berglund H Hard T Rapid screening for improved solubility of small human proteins produced as fusion proteins in Escherichia coli Protein Sci 2002 11 2 313 321 11790841 10.1110/ps.22102 PMC2373440

[B41] Shih YP Kung WM Chen JC Yeh CH Wang AH Wang TF High-throughput screening of soluble recombinant proteins Protein Sci 2002 11 7 1714 1719 12070324 10.1110/ps.0205202 PMC2373646

[B42] Eshaghi S Hedren M Nasser MI Hammarberg T Thornell A Nordlund P An efficient strategy for high-throughput expression screening of recombinant integral membrane proteins Protein Sci 2005 14 3 676 683 10.1110/ps.041127005 15689514 PMC2279288

[B43] Kapust RB Waugh DS Escherichia coli maltose-binding protein is uncommonly effective at promoting the solubility of polypeptides to which it is fused Protein Sci 1999 8 8 1668 1674 10.1110/ps.8.8.1668 10452611 PMC2144417

[B44] Dyson MR Shadbolt SP Vincent KJ Perera RL McCafferty J Production of soluble mammalian proteins in Escherichia coli: identification of protein features that correlate with successful expression BMC Biotechnol 2004 4 32 10.1186/1472-6750-4-32 15598350 PMC544853

[B45] Cabrita LD Dai W Bottomley SP A family of E. coli expression vectors for laboratory scale and high throughput soluble protein production BMC Biotechnol 2006 6 12 10.1186/1472-6750-6-12 16509985 PMC1420288

[B46] Korf U Kohl T van der Zandt H Zahn R Schleeger S Ueberle B Wandschneider S Bechtel S Schnolzer M Ottleben H Large-scale protein expression for proteome research Proteomics 2005 5 14 3571 3580 10.1002/pmic.200401195 16127724

[B47] Luan CH Qiu S Finley JB Carson M Gray RJ Huang W Johnson D Tsao J Reboul J Vaglio P High-throughput expression of C. elegans proteins Genome Res 2004 14 10B 2102 2110 10.1101/gr.2520504 15489332 PMC528926

[B48] Studier FW Use of bacteriophage T7 lysozyme to improve an inducible T7 expression system J Mol Biol 1991 219 1 37 44 10.1016/0022-2836(91)90855-Z 2023259

[B49] Fox JD Routzahn KM Bucher MH Waugh DS Maltodextrin-binding proteins from diverse bacteria and archaea are potent solubility enhancers FEBS Lett 2003 537 1-3 53 57 10.1016/S0014-5793(03)00070-X 12606030

[B50] Baneyx F Recombinant protein expression in Escherichia coli Curr Opin Biotechnol 1999 10 5 411 421 10.1016/S0958-1669(99)00003-8 10508629

[B51] Ignatova Z Mahsunah A Georgieva M Kasche V Improvement of posttranslational bottlenecks in the production of penicillin amidase in recombinant Escherichia coli strains Appl Environ Microbiol 2003 69 2 1237 1245 10.1128/AEM.69.2.1237-1245.2003 12571052 PMC143610

[B52] Perez-Perez J Marquez G Barbero JL Gutierrez J Increasing the efficiency of protein export in *Escherichia coli* Biotechnology (N Y) 1994 12 2 178 180 10.1038/nbt0294-178 7764432

[B53] Huang X Miller W A time-efficient, linear-space local similarity algorithm Adv Appl Math 1991 12 337 357 10.1016/0196-8858(91)90017-D

[B54] Tsunoda Y Sakai N Kikuchi K Katoh S Akagi K Miura-Ohnuma J Tashiro Y Murata K Shibuya N Katoh E Improving expression and solubility of rice proteins produced as fusion proteins in *Escherichia coli* Protein Expr Purif 2005 42 2 268 277 10.1016/j.pep.2005.04.002 15914031

[B55] Pines O Inouye M Expression and secretion of proteins in E. coli Mol Biotechnol 1999 12 1 25 34 10.1385/MB:12:1:25 10554771

[B56] Humphreys DP Sehdev M Chapman AP Ganesh R Smith BJ King LM Glover DJ Reeks DG Stephens PE High-level periplasmic expression in Escherichia coli using a eukaryotic signal peptide: importance of codon usage at the 5' end of the coding sequence Protein Expr Purif 2000 20 2 252 264 10.1006/prep.2000.1286 11049749

[B57] Swartz JR Advances in Escherichia coli production of therapeutic proteins Curr Opin Biotechnol 2001 12 2 195 201 10.1016/S0958-1669(00)00199-3 11287237

[B58] Bessette PH Aslund F Beckwith J Georgiou G Efficient folding of proteins with multiple disulfide bonds in the Escherichia coli cytoplasm Proc Natl Acad Sci USA 1999 96 24 13703 13708 10.1073/pnas.96.24.13703 10570136 PMC24128

[B59] Liddy N Molloy PE Bennett AD Boulter JM Jakobsen BK Li Y Production of a soluble disulfide bond-linked TCR in the cytoplasm of Escherichia coli trxB gor mutants Mol Biotechnol 2010 45 2 140 149 10.1007/s12033-010-9250-0 20143183

[B60] Peisley AA Gooley PR High-level expression of a soluble and functional fibronectin type II domain from MMP-2 in the Escherichia coli cytoplasm for solution NMR studies Protein Expr Purif 2007 53 1 124 131 10.1016/j.pep.2006.12.005 17251038

[B61] Jurado P de Lorenzo V Fernandez LA Thioredoxin fusions increase folding of single chain Fv antibodies in the cytoplasm of Escherichia coli: evidence that chaperone activity is the prime effect of thioredoxin J Mol Biol 2006 357 1 49 61 10.1016/j.jmb.2005.12.058 16427080

[B62] Dobrovetsky E Lu ML Andorn-Broza R Khutoreskaya G Bray JE Savchenko A Arrowsmith CH Edwards AM Koth CM High-throughput production of prokaryotic membrane proteins J Struct Funct Genomics 2005 6 1 33 50 10.1007/s10969-005-1363-5 15909233

[B63] Cappuccio JA Blanchette CD Sulchek TA Arroyo ES Kralj JM Hinz AK Kuhn EA Chromy BA Segelke BW Rothschild KJ Cell-free co-expression of functional membrane proteins and apolipoprotein, forming soluble nanolipoprotein particles Mol Cell Proteomics 2008 7 11 2246 2253 10.1074/mcp.M800191-MCP200 18603642 PMC2577204

[B64] Klammt C Schwarz D Fendler K Haase W Dotsch V Bernhard F Evaluation of detergents for the soluble expression of alpha-helical and beta-barrel-type integral membrane proteins by a preparative scale individual cell-free expression system Febs J 2005 272 23 6024 6038 10.1111/j.1742-4658.2005.05002.x 16302967

[B65] Gardy JL Spencer C Wang K Ester M Tusnady GE Simon I Hua S deFays K Lambert C Nakai K PSORT-B: Improving protein subcellular localization prediction for Gram-negative bacteria Nucleic Acids Res 2003 31 13 3613 3617 10.1093/nar/gkg602 12824378 PMC169008

[B66] Gatlin CL Pieper R Huang ST Mongodin E Gebregeorgis E Parmar PP Clark DJ Alami H Papazisi L Fleischmann RD Proteomic profiling of cell envelope-associated proteins from Staphylococcus aureus Proteomics 2006 6 5 1530 1549 10.1002/pmic.200500253 16470658

[B67] Gill SC von Hippel PH Calculation of protein extinction coefficients from amino acid sequence data Anal Biochem 1989 182 2 319 326 10.1016/0003-2697(89)90602-7 2610349

